# A neural basis for the visual sense of number and its development: A steady-state visual evoked potential study in children and adults

**DOI:** 10.1016/j.dcn.2017.02.011

**Published:** 2017-03-02

**Authors:** Joonkoo Park

**Affiliations:** Department of Psychological and Brain Sciences, Commonwealth Honors College, University of Massachusetts, 135 Hicks Way, Amherst, MA 01003, United States

**Keywords:** Numerosity, Steady-state visual evoked potential, Child development, Visual cortex, Approximate number system

## Abstract

While recent studies in adults have demonstrated the existence of a neural mechanism for a visual sense of number, little is known about its development and whether such a mechanism exists at young ages. In the current study, I introduce a novel steady-state visual evoked potential (SSVEP) technique to objectively quantify early visual cortical sensitivity to numerical and non-numerical magnitudes of a dot array. I then examine this neural sensitivity to numerical magnitude in children between three and ten years of age and in college students. Children overall exhibit strong SSVEP sensitivity to numerical magnitude in the right occipital sites with negligible SSVEP sensitivity to non-numerical magnitudes, the pattern similar to what is observed in adults. However, a closer examination of age differences reveals that this selective neural sensitivity to numerical magnitude, which is close to absent in three-year-olds, increases steadily as a function of age, while there is virtually no neural sensitivity to other non-numerical magnitudes across these ages. These results demonstrate the emergence of a neural mechanism underlying direct perception of numerosity across early and middle childhood and provide a potential neural mechanistic explanation for the development of humans’ primitive, non-verbal ability to comprehend number.

## Highlights

• A neural basis for direct perception of number in children (3–10 y) was tested.
• Novel SSVEP method assessed numerical and non-numerical magnitude processing.
• SSVEPs were modulated exclusively by numerosity and not by non-numerical cues.
• Selective SSVEP sensitivity to numerosity increased as a function of age.
• Findings illustrate the emergence of a neural mechanism for the visual number sense.

## 1 Introduction

Recent studies in adults have demonstrated the existence of a neurocognitive mechanism for a visual sense of number (Burr and Ross, 2008, Harvey et al., 2013, Park et al., 2016). For instance, perceived numerosity of a dot array is susceptible to adaptation, lending support to the view that the visual system processes numerosity like a primary visual property that is not reducible to other non-numerical magnitudes (Burr and Ross, 2008; but see Durgin, 1995, Durgin, 2008). In fact, numerical information of a dot array is processed extremely early in the cortical visual stream prior to the processing of, if any, non-numerical cues of the array (Park et al., 2016). Such findings indicating a neural mechanism for direct perception of number are consistent with the idea that our non-verbal ability to comprehend number (based on the so-called approximate number system, ANS) is developmentally and evolutionarily primitive (Gallistel and Gelman, 1992, Dehaene, 1999, Spelke and Kinzler, 2007). Nevertheless, little is known about the developmental trajectory of the neural mechanism for direct perception of number in young children.

Previous behavioral and neuroimaging studies provide some hints to the existence of the visual sense of number from young ages. For instance, when given a numerosity comparison task in which participants are asked to choose one of two briefly presented dot arrays that contains more dots, children as young as three years old reliably differentiate two different numerosities in a 3:4 ratio, and this ability improves dramatically in early childhood (Halberda and Feigenson, 2008, Piazza et al., 2010, Halberda et al., 2012, Mussolin et al., 2012). At the neural level, functional magnetic resonance imaging (fMRI) and electroencephalogram (EEG) studies using a passive viewing paradigm have shown that infants and children’s brain, particularly in the posterior parietal cortex or more broadly in the dorsal stream, is sensitive to changes in numerosity of dot arrays (Cantlon et al., 2006, Izard et al., 2008, Hyde and Spelke, 2011, Libertus et al., 2011).

Findings from such previous studies themselves, however, do not provide firm evidence for direct perception of number. Numerosity of a dot array covaries with other non-numerical properties, such as the surface area or density of the array. Therefore, it is difficult to distinguish whether children’s behavioral or neural response is based on numerosity per se or on other non-numerical cues of the array. For example, a closer look into children’s behavioral patterns reveals that their performance is often near chance when numerosity is incongruent with non-numerical perceptual cues (Rousselle et al., 2004, Halberda and Feigenson, 2008, Soltesz et al., 2010). Thus, it is difficult to distinguish whether the performance in a numerosity comparison task represents the precision of numerosity representation or one’s ability to extract numerical information amidst non-numerical cues (or both) (see Rousselle et al., 2004, Rousselle and Noël, 2008, Soltesz et al., 2010, Defever et al., 2013, Fuhs and McNeil, 2013, Gilmore et al., 2013). Such findings also speak to the importance of investigating the representation of non-numerical magnitudes in order to better understand the representation of numerical magnitudes (as argued in Leibovich and Henik, 2013).

**Fig. 1:**
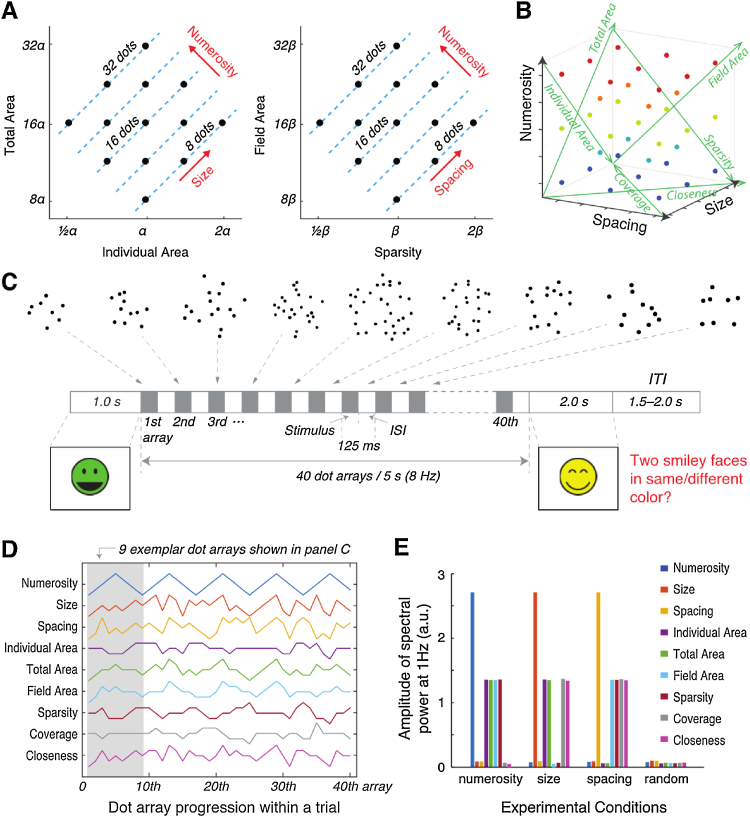
Stimuli and experimental procedure. (A) This panel illustrates the relationship between numerosity, individual area, total area, sparsity, and field area. Dot array stimuli were systematically constructed to cover equal ranges of these dimensions of interest. Logarithmic scaling allowed a construction of two orthogonal dimensions to numerosity: size and spacing. The two axes are two 2-dimensional projections of a 3-dimensional parameter space. (B) A 3-dimensional view of the parameter space. For a better visualization, the parameter points where dot arrays were sampled from were color-coded so that each level of numerosity is shown in the same color. Green axes illustrate the dimensions of individual area, total area, field area, sparsity, coverage, and closeness, each of which can be expressed as a linear combination of numerosity, size, and spacing (see Table 1). (C) This panel illustrates the progression of a trial. On each trial, 40 dot arrays were presented for 5 s (rate of 8 Hz) between the two smiley faces. Participants were asked to judge whether the two face colors matched or not and were told to focus on the screen while the dot arrays flickered. The 40 dot arrays fluctuated in a systematic way along one of the three dimensions (numerosity, size, and spacing) or in a random manner. This particular example shows a systematic variation in numerosity, and the first through the ninth exemplary dot arrays are illustrated. (D) An example of a dot array progression within a trial as a function of the dimensions of interest. This particular trial illustrates a case where numerosity varied systematically at 1 Hz (as shown in panel C). (E) 1-Hz spectral magnitude of the dimensions of interest under four hypothetical experimental conditions. These values are mean single-sided amplitude spectrum (in arbitrary unit, a.u.) across all the participants computed empirically from the all the dot arrays images that participants viewed. Thus, if the measured EEG is sensitive, for example, to numerosity, then 1-Hz spectral magnitude should be large in the numerosity condition and small in all other conditions.

The present study investigates the developmental trajectory of the neural mechanisms underlying the perception of numerical and non-numerical magnitudes, thereby testing the existence and the development of a neural mechanism for the visual sense of number. The study builds on a previously developed technique that quantifies neural sensitivity to numerical and non-numerical magnitudes of a dot array (Park et al., 2016, Fornaciai and Park, 2017). The essence of that technique is to systematically construct dot arrays spanning in three orthogonal dimensions: numerosity, size and spacing (see Fig. 1A & B) (see also DeWind et al., 2015 for a behavioral study). Conceptually, size refers to the dimension that varies in the surface area of a dot array while holding numerosity constant, and spacing refers to the dimension that varies in overall inter-dot distance of the array while holding numerosity constant. A regression-based analysis is then used to quantify the modulation of neural activity by the three orthogonal dimensions and linear combinations of the three dimensions, thus allowing the evaluation of numerical and non-numerical representations in the brain. Critically, this neural approach allows a passive viewing paradigm in which participants are not instructed to focus on any magnitude dimension, thus enabling an objective measurement of numerical and non-numerical representations without any biases induced by the task. In a previous event-related potential (ERP) study in adults (Park et al., 2016), two distinct processing stages early in the visual stream (∼75 ms in the medial occipital site; ∼180 ms in the bilateral occipital site) showed selective encoding of numerosity and very little, if any, representation of non-numerical magnitudes.

Here, I introduce a novel approach based on the steady-state visual evoked potential (SSVEP) (Regan, 1980, Morgan et al., 1996, Srinivasan et al., 1999, Vialatte et al., 2010, Norcia et al., 2015), while adopting the previously developed analytic technique (DeWind et al., 2015, Park et al., 2016), in order to quantify the neural sensitivity to numerical and non-numerical magnitudes of a dot array (see Materials and methods). The use of the SSVEPs (as opposed to the ERPs used in Park et al., 2016) makes it more feasible to study young children because SSVEPs are known to have higher signal-to-noise ratio and to be more robust to artifacts (see Vialatte et al., 2010). In a cross-sectional design, this SSVEP method was used to quantify 3- to 10-year-old children’s neural sensitivity to numerical and non-numerical magnitudes. This age range was selected because the acuity of the ANS (mostly computed from a numerosity comparison task) improves dramatically in the first decade of life (Halberda and Feigenson, 2008, Mussolin et al., 2012); therefore, children’s magnitude perception is expected to undergo a major change in this period. I also tested adult participants using the virtually identical paradigm partly in order to verify the validity of this SSVEP approach and to provide a comparison between children and adults’ neural sensitivity to numerical and non-numerical magnitudes. On the one hand, I hypothesized a robust neural sensitivity to numerosity and relatively weak sensitivity to non-numerical magnitudes in children, based on the theoretical claim that our number sense is developmentally and evolutionarily primitive (Gallistel and Gelman, 1992, Dehaene, 1999) as well as on the previous finding in adults (Park et al., 2016). On the other hand, it was an open question as to how the neural representation for numerosity might change over development.

## 2 Materials and methods

### 2.1 Participants

Fifty-two children between ages of 3 years 0 months and 10 years 11 months were initially recruited from the local community around the University of Massachusetts Amherst. Of these children, three children refused to participate or quit during experiment setup, and two children were removed from the analysis due to excessive movement during the entire EEG session, leaving a total of 47 children in the final sample (29 girls). The distribution of the age was as follows: 6 children (3 ≤ age < 5), 15 children (5 ≤ age < 7), 15 children (7 ≤ age < 9), and 11 children (9 ≤ age < 11). All children spoke English as their first language and were Caucasian. The median education level of the mothers was master’s degree and of fathers was bachelor’s degree. In addition to child participants, 40 college-student volunteers (9 male; aged 18.4–23.1 years) participated from the departmental subject pool at the University of Massachusetts Amherst.

All participants were right-handed, had normal or corrected-to-normal vision, had no history of developmental disability, and were neurologically intact (screened by self or parental reports). Families of child participants were compensated $15 for their time and transportation, and children were given a choice of a toy prize at the end of the study. The college-student participants were given departmental class credit for their participation in the study. All procedures were approved by the University of Massachusetts Institutional Review Board.

### 2.2 Stimuli

Visual stimuli were black dot arrays on a gray background (RGB values of 170). Each dot array was unique. Dots were homogeneous in size within an array and were drawn within an invisible circular field. The minimum distance between any two dots (from the edge of one dot to the edge of another) was at least a third of the dot diameter.

Following the previously developed design (DeWind et al., 2015, Park et al., 2016, Fornaciai and Park, 2017), numerical and various non-numerical dimensions of the dot arrays varied systematically in relation to one other in a log scale. They also varied in equal ranges, in which the maximum value of a dimension was four times of its minimum value. The numerosity used in this study was 8, 11, 16, 23 or 32 dots in an array (5 equidistant levels in a log-base-2 scale). Non-numerical dimensions primarily used to construct the stimulus space included individual area, total area, field area, and sparsity (see Table 1 for their definitions). After a logarithmic scaling, two dimensions orthogonal to the dimension of numerosity were identified (Fig. 1A). Note that logarithmic scaling abides the Weber-Fechner law, as perceived intensity is proportional to the log of stimulus intensity. The first of the two, named size, represented the dimension that changes both individual area and total area concurrently when numerosity is held constant. Mathematically, it is expressed as log(size) = log(individual area) + log(total area). The second of the two, named spacing, represented the dimension that changes both field area and sparsity concurrently when numerosity is held constant. Mathematically, it is expressed as log(spacing) = log(field area) + log(sparsity). Importantly, numerosity, size, and spacing could be manipulated independently. For instance, 16 dots could be drawn either with larger dots or with smaller dots. At the same time, the same 16 dots could be scattered widely in a large invisible circular area or packed in a condensed invisible circular area. The orthogonal nature of the three dimensions is depicted by the 3D parameter space illustrated in Fig. 1B. Crucially, this unique stimulus design allows the representation of all possible non-numerical dimensions as a linear combination of log(numerosity), log(size), and log(spacing), as long as the dimension can be expressed as a function of numerosity (n), radius of the individual dot (r_d_), and the radius of the invisible circular field in which the dots are drawn (r_f_). For instance, with simple algebra, log of total perimeter, n × 2πr_d_, can be expressed as a linear combination of log(numerosity) and log(size) (see Table 1). In addition, a linear combination of size and spacing identified the dimension represented by the total area divided by the field area, named coverage. Likewise, another linear combination of size and spacing represented the overall scaling of the dots, named closeness. Table 1 describes how various non-numerical dimensions are represented as a linear combination of log(numerosity), log(size), and log(spacing).

**Table 1:** Relations between numerical and various non-numerical dimensions.

Dimension	Dimension as a function of n, r_d_, and r_f_	Log of dimension as a function of log(numerosity), log(size), and log(spacing)
Individual area	πr_d_^2^	½log(size)-½log(numerosity)
Total area	n × πr_d_^2^	½log(size) + ½log(numerosity)
Field area	πr_f_^2^	½log(spacing) + ½log(numerosity)
Sparsity	πr_f_^2^/n	½log(spacing)-½log(numerosity)
Individual perimeter	2πr	log(2√π) + ¼log(size)-¼log(numerosity)
Total perimeter	n × 2πr	log(2√π) + ¼log(size) + ¾log(numerosity)
Coverage	n × r_d_^2^/r_f_^2^	½log(size)-½log(spacing)
Closeness	π^2^·r_d_^2^·r_f_^2^	½log(size) + ½log(spacing)

Note: n = numerosity, r_d_ = radius of individual dot, and r_f_ = radius of the invisible circular field in which the dots are drawn.

The minimum individual area was ∼78.5 pixel^2^ encompassing 0.177° (10 pixels) in diameter, and the maximum individual area was ∼314.2 pixel^2^ encompassing 0.354° (20 pixels) in diameter. The minimum field area was ∼25,447 pixel^2^ encompassing 3.18° (180 pixels) in diameter, and the maximum field area was ∼101,787 pixel^2^ encompassing 6.37° (360 pixels) in diameter. Note that all of the dimensions of interest ranged in a four-fold difference from their minimum to maximum values.

The basic idea behind the SSVEP method is to present stimuli in a specific temporal frequency in order to “tag” a neural activity pattern to the occurrence of those stimuli (Regan, 1980, Vialatte et al., 2010). Each trial of the SSVEP experiment contained 5-s long presentations of a total of 40 dot arrays at a rate of 8 Hz. There were four experimental conditions where dot arrays were systematically varied in (1) numerosity, (2) size, (3) spacing, or (4) at random.

In the numerosity condition, 40 dot arrays were drawn so that there was a 1-Hz variation only in numerosity. For instance, the first dot array was drawn to contain 11 dots while its size and spacing parameters were randomly chosen. Then, the second dot array was drawn to contain 16 dots while its size and spacing parameters were randomly chosen. The third dot array was drawn to contain 23 dots, the fourth to contain 32 dots, the fifth to contain 23 dots, and so on. In other words, throughout the course of a trial, numerosity was chosen to fluctuate between its lowest and highest values while other parameters were chosen randomly (Fig. 1D). The starting value of each trial was randomized across trials—for instance, some trials started its 5-s long presentation with 8 dots while others started with 23 dots. This phase randomization was intended to prevent participants from consistently seeing the same value as the first dot array of all the trials. Overall, this systematic manipulation resulted in a 1-Hz fluctuation in numerosity (Fig. 1D & E). Thus, the spectral power at 1 Hz under this experimental condition is thought to be arising from a systematic fluctuation in numerosity. Note, however, that a unit change (in log scale) in numerosity is associated with a half-unit change in individual area, total area, field area, and sparsity. At the same time, one unit change in numerosity is associated with a quarter-unit change in individual perimeter, and one unit change in numerosity is associated with a three-quarters-unit change in total perimeter (see Table 1). Thus, a systematic fluctuation in numerosity results in some spectral magnitude in those other non-numerical dimensions. For example, if numerosity fluctuates at 1 Hz with a power of 100, the power of 1 Hz fluctuation in individual area, total area, field area, and sparsity is 50, the power of 1 Hz fluctuation in individual perimeter is 25, and the power of 1 Hz fluctuation in total perimeter is 75 (see Fig. 1E). Therefore, in order to assess whether 1-Hz SSVEP magnitude is arising solely from sensitivity to numerosity or from sensitivity to other non-numerical cues, one needs to consider other experimental conditions where systematic 1-Hz fluctuation is in size or spacing.

The size and spacing conditions were identical to the scheme described above for the numerosity condition, except systematic fluctuation was along the size and spacing dimensions, respectively. This manipulation resulted in the variation of size and spacing at a rate of 1 Hz, with moderate spectral magnitude for the dimensions that are correlated with size (individual area, total area, individual perimeter, total perimeter, coverage, and closeness) and spacing (field area, sparsity, coverage, closeness) (see Fig. 1E). Thus, 1-Hz SSVEP magnitudes from the three experimental conditions all together allow one to tease apart the source of neural frequency tagging.

In the fourth experimental (random) condition, 40 dot arrays were chosen completely randomly across the stimulus parameter space. The stimuli were displayed on a 24-inch ultralow latency monitor set to a 144 Hz refresh rate.

### 2.3 Task and procedure

The SSVEP experiment involved participants to passively view a series of dot arrays (as described above in *Stimuli*) while a secondary—color judgment—task was given to ensure that participants paid attention to the screen (see Fig. 1C). Each trial began with an open-mouthed smiley face with one of six colors (red, yellow, green, cyan, blue, purple) presented on the center of the screen for 1000 ms. This smiley was then followed by a 5-s long dot array presentation, during which a total of 40 dot arrays were presented. Each dot array and a white fixation cross were centrally presented for 62.5 ms, which was then followed by an interstimulus interval of another 62.5 ms where only the white fixation cross was presented. Thus, each dot array appeared every 125 ms for a total of 5 s (8 Hz; a photosensor mounted on a corner of the presentation monitor was used to ensure this stimulus presentation rate). After the last dot array and its subsequent interstimulus interval, a colored close-mouthed smiley appeared centrally for 2000 ms (1500 ms in adults participants), during which a response was accepted. The color of this second smiley was either identical (50%) or not to the first smiley. The task was to press a joypad button under the right index finger if the color matched or to press a button under the left index finger if not. Button assignment was counterbalanced across participants. The response was followed by an intertrial interval chosen randomly from a uniform distribution between 1500 and 2000 ms.

For children, each block consisted of 20 trials, which took about three minutes. Thus, each of the four conditions (numerosity, size, spacing, and random) appeared five times in each block. Children were encouraged to perform as many blocks as possible with the goal of 6 blocks for children of age ≤ 6 years and 8 blocks for children of age ≥ 7 years. On average, 3–6 year-old children performed 6 blocks and 7–10 year-old children performed 9 blocks. For adults, each block consisted of 40 trials (thus twice as long as the children’s block), and they performed a total of six blocks. All participants received a few practice trials (until they were able to respond correctly to the color judgment task in at least two trials) prior to performing the real experiment with EEG recordings.

Until the end of the experiment, participants were not told about the goal of the study nor anything about the magnitude or quantity aspect of the stimuli. At the end of the experiment, some adult participants were verbally prompted to report what they thought of the flashing dots and whether they noticed any differences in the pattern of dot flashing across different trials. None of the participants were aware of the experimental manipulations and none were even close to thinking that *magnitude* was key to the experiment.

### 2.4 Electrophysiological recording and analysis

In a dimly lit, quiet room, the electroencephalogram (EEG) was recorded continuously from 64 channels mounted in an extended coverage, triangulated equidistant cap (M10, EasyCap, GmbH) using a low-pass filter of 100 Hz at a sampling rate of 1000 Hz (actiCHamp, Brain Products, GmbH). All channels were referenced to the vertex (Cz) during recording. The electro-oculogram (EOG) was monitored with electrodes below the left eye and just lateral to the left and right canthi. Impedances of all channels were below 35 kΩ (below 25 kΩ in adult participants). Slightly higher impedance was tolerated in children for time efficiency, possibly accompanied by a sacrifice in data quality. However, according to Kappenman and Luck (2010), it is unlikely that these small differences in impedance threshold affect data quality.

EEG data were processed offline in Matlab R2013a using the EEGLAB software package (Delorme and Makeig, 2004), the associated ERPLAB toolbox (Lopez-Calderon and Luck, 2014), and custom scripts. The continuous EEG data were re-referenced to the average of all channels and were high-pass filtered at 0.1 Hz. In order to phase-lock data to the relevant dimension (numerosity, size, or spacing) and to avoid the initial transient response to the onset of the dot array, 4-s-long EEG epochs were extracted that were time-locked to the onset of the presentation of the highest value in the corresponding condition (numerosity, size, or spacing) closest to 500 ms after dot array onset.

A parallel, independent pipeline of analysis was performed with nearly identical parameters, except the epochs covered from −1000 to 6000 ms time-locked to the dot array onset. Independent component analysis was used on these epochs to identify component weights associated with eye blinks and movements. Note that a longer (7-s) epoch was used capture eye blink and movement patterns in a more exhaustive time range including before and after the dot array presentation. The independent component weights were then used to correct for such artifacts in the main analysis pipeline with 4-s-long epochs. Subsequent analyses were done on those artifact-corrected 4-s-long epochs.

Visual inspection of the epochs revealed extreme data points in some channels, particularly in children’s data likely due to movement. Thus, channels with the difference between maximum and minimum amplitude exceeding more than 5000 μV within an epoch were spherically interpolated on the epoch-by-epoch basis. Note that the channels with these extreme artifacts were interpolated instead of rejected because rejecting would lead to too many epochs rejected even if the data in the channels of interest are free of such an artifact. Importantly, interpolation of the extreme artifacts, which is assumed to occur at random, does not bias the results towards any of the four experimental conditions tested. Approximately 0.005% of all the epochs in all the children’s data were interpolated. Using the same criterion, less than 0.001% of all the epochs in the adults’ data were interpolated. After correcting for these extreme artifacts, epochs in which peak-to-peak amplitude exceeding 250 μV (which is improbable for this study) at the five posterior channels (at or near PO7, O1, Oz, O2, and PO8) were rejected. These channels of interest were selected based on the previous finding that demonstrated a strong effect of ERP modulation by dot arrays (see Fig. 3 of Park et al., 2016). The mean artifact rejection rate was 13.5% in children and 8.10% in adults.

Finally, selective averaging of the epochs was performed for each of the four conditions, and the spectral magnitude (single-sided amplitude spectrum) during the 4-s long epoch was computed using fast Fourier transformation (FFT) analysis for each of the four experimental conditions. Greenhouse-Geisser corrected *p*-values were reported when violation of sphericity in a repeated-measures ANOVA was expected.

The SSVEP analysis concerned spectral magnitudes at two frequencies: 8 Hz and 1 Hz. The dot arrays were presented at 8 Hz; therefore, 8-Hz SSVEPs represent neural responses tagged by the flickering of the dot arrays potentially agnostic to any experimental manipulations regarding systematic variations in numerosity, size, or spacing. At the same time, there existed a systematic fluctuation in numerosity, size, or spacing at 1 Hz; therefore, 1-Hz SSVEPs represent neural sensitivity to changes in numerosity, size, or spacing, with smaller effects of other quantity dimensions that are correlated with each of them (see Fig. 1E). Finally, 1-Hz SSVEPs in the random condition represent a baseline SSVEP response that is not associated with any magnitude representations.

## 3 Results

I first report the results in adults to demonstrate the validity of the SSVEP approach in comparison to the previous ERP results (Park et al., 2016). I then report the results in children to assess the developmental trajectory of the neural representations for numerical and non-numerical magnitudes.

### 3.1 Adults show selective SSVEP sensitivity to numerical magnitude

#### 3.1.1 Behavioral results

The mean (±std) accuracy of the color judgment task was 0.952 (±0.0445) and the mean (±std) response time was 649.4 (±122.8) ms.

#### 3.1.2 SSVEP response to the dot arrays in adults

**Fig. 2:**
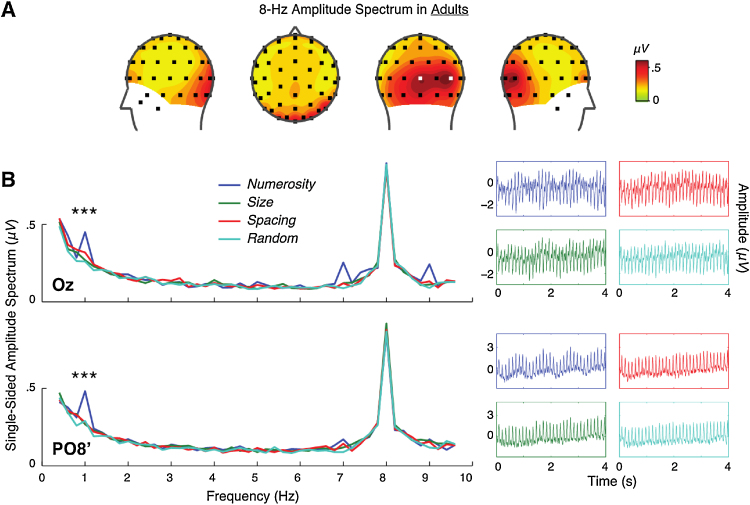
SSVEP results in adults. (A) Topographic distribution of 8-Hz spectral magnitude collapsed across all four experimental conditions. Oz and PO8′ are indicated in white squares in the posterior perspective. (B) Spectral magnitude as a function of frequency. Note that 8 Hz represents the frequency at which the dot arrays were presented, and 1 Hz represents the frequency at which dot arrays fluctuated in numerosity, size, or spacing. Asterisks (***) indicate significant (p < 0.001) within-subjects effects of experiment condition. Average raw EEG signal across subjects for each experimental condition is also presented for visual inspection purposes.

I examined the spectral magnitude at 8 Hz across all the channels to identify electrode sites that are most responsive to dot array flickering frequency regardless of the four experimental conditions. As illustrated in Fig. 2A, the topographic distribution of 8-Hz spectral magnitude peaked at the medial (Oz) and right (PO8′) occipital channels. This right occipital channel was closest but slightly lateral (∼0.16 rad) to PO8 in the standard 10–20 system (henceforth referred to as PO8′) in this paper.

**Fig. 3:**
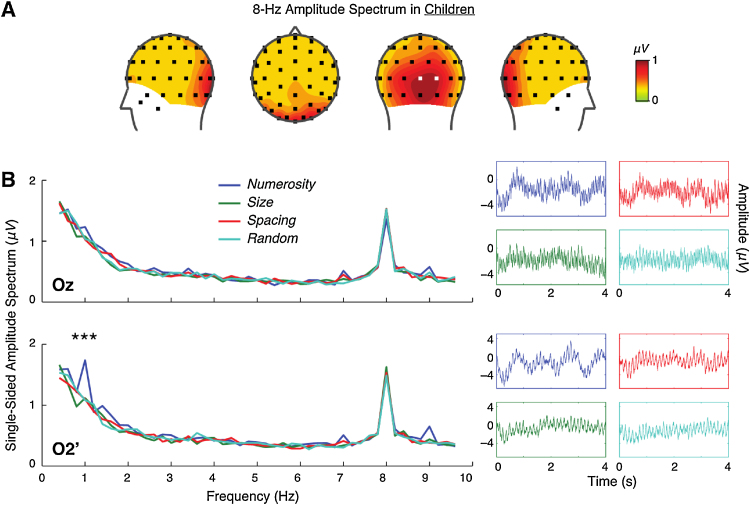
SSVEP results in children. (A) Topographic distribution of 8-Hz spectral magnitude collapsed across all four experimental conditions. Oz and O2′ are indicated in white squares in the posterior perspective. (B) Spectral magnitude as a function of frequency. Note that 8 Hz represents the frequency at which the dot arrays were presented, and 1 Hz represents the frequency at which dot arrays fluctuated in numerosity, size, or spacing. Asterisks (***) indicate significant (p < 0.001) within-subjects effects of experiment condition. Average raw EEG signal across subjects for each experimental condition is also presented for visual inspection purposes.

Because SSVEPs do not contain temporal information, it is difficult to evaluate which processing stage is responsible for such 8-Hz peaks. Nevertheless, it is noteworthy to point out that these locations (Oz and PO8′) are consistent with a previous finding (Park et al., 2016) that showed maximal sensitivity to manipulations across all quantity dimensions (see Figs. 3 and 6B in Park et al., 2016). In particular, the ERPs were maximally sensitive to dot arrays at Oz around 75 ms (the C1 ERP component, Clark et al., 1994, Di Russo et al., 2002) and near PO7 and PO8 around 180 ms after the dot array onset (between N1 and P2 ERP components). Accordingly, it is reasonable to speculate that the present 8-Hz SSVEP signature over these occipital sites arises from C1 and P2 components in the medial and right occipital sites, respectively. Note, however, that unlike the previous study (Park et al., 2016), the 8-Hz spectral magnitude did not peak near PO7 in the current SSVEP experiment, perhaps indicating that the neural source generating the oscillatory power over the left occipitoparietal site is not strong enough to be captured by SSVEPs. Thus, subsequent analyses were done at channels Oz and PO8′.

I tested whether the 8-Hz spectral magnitude differed across the four conditions (numerosity, size, spacing, and random) in the two channels (Oz and PO8′) using a two-way repeated-measures ANOVA. The interaction between condition and channel (F(3, 117) = 1.215, p = 0.300), the main effect of condition (F(3, 117) = 0.808, p = 0.475), and the main effect of channel were not close to statistical significance (F(1, 39) = 0.012, p = 0.912). These results indicate that systematic variations in the dimensions of interest did not affect the spectral magnitude at the frequency rate (8 Hz) at which the dot arrays flickered. The two channels that peaked in 8-Hz spectral magnitude, Oz and PO8′, were assumed to be most responsive to dot array presentations; therefore, those two channels were chosen for further investigations in the systematic variations in numerosity, size, and spacing, as reported in the following Section.

#### 3.1.3 SSVEP index of numerical sensitivity in adults

SSVEP sensitivity to numerosity, size, and spacing was assessed by quantifying the spectral magnitude at 1 Hz over Oz and PO8′. As shown in Fig. 2B, there was a robust enhancement of spectral magnitude at 1 Hz in both channels predominately in the numerosity condition compared to all other conditions. The effects of spectral magnitude as a function of experimental conditions (numerosity, size, spacing, and random) were quantified using a repeated-measures ANOVA separately at Oz and PO8′.

At Oz, there was a significant effect of condition (F(3, 117) = 8.891, p < 0.001) with a within-subjects effect size of η_p_^2^ = 0.186. Moreover, a contrast analysis revealed that the spectral magnitude in the numerosity condition was significantly greater than that in the random condition (F(1, 39) = 15.358, p < 0.001), which suggests that the brain is sensitive to numerosity. However, as some non-numerical dimensions are inherently correlated with numerosity (see Fig. 1A; Table 1), systematic 1-Hz fluctuation in numerosity also results in some degree of 1-Hz fluctuation in those other dimensions (see *2.2 Stimuli*; Fig. 1E). Therefore, a robust 1-Hz spectral magnitude in the numerosity condition may be partially driven by moderate 1-Hz fluctuations in those other dimensions.

Nevertheless, contrast analyses further revealed that the 1-Hz spectral magnitude was significantly greater in the numerosity condition than that in the size (F(1, 39) = 15.549, p < 0.001) and spacing (F(1, 39) = 9.457, p = 0.004) conditions. Furthermore, the 1-Hz spectral magnitude for size and spacing did not differ from the spectral magnitude in the random condition (p > 0.142). Such patterns were critical in ruling out alternative explanations. First, individual area, total area, and individual perimeter are equally associated with both numerosity and size (see Table 1; Fig. 1E). Thus, substantially greater 1-Hz spectral magnitude for numerosity than for size falsifies the hypothesis that 1-Hz spectral magnitude for numerosity is driven by individual area, total area, or individual perimeter (otherwise, 1-Hz spectral magnitude for size should be comparable to that for numerosity). Moreover, if individual area, total area, or individual perimeter has any effect on the SSVEPs, the 1-Hz spectral magnitude for size should have been reliably greater than the spectral magnitude in the random condition. That was not the case. Second, field area and sparsity are equally associated with both numerosity and spacing (see Table 1; Fig. 1E). Thus, substantially greater 1-Hz spectral magnitude for numerosity than for spacing falsifies the hypothesis that 1-Hz spectral magnitude for numerosity is driven by field area or sparsity (otherwise, 1-Hz spectral magnitude for spacing should be comparable to that for numerosity). In addition, no reliable difference in the 1-Hz spectral magnitude between the spacing and the random conditions suggests that field area or sparsity has no effects on the SSVEPs. Third, total perimeter is more correlated with numerosity than it is correlated with size (see Table 1; Fig. 1E); therefore, one may wonder if the resulting 1-Hz spectral magnitude by numerosity could reflect neural sensitivity to total perimeter rather than numerosity. However, if total perimeter were driving the current SSVEP results, then 1-Hz spectral magnitude should have been substantially greater both in the numerosity and the size conditions than in the random condition. That was not the case. In fact, if this were the case, then the 1-Hz spectral magnitude for size should have been at least a third of the 1-Hz spectral magnitude for numerosity.

Likewise at PO8′, there was a significant effect of condition (F(3, 117) = 16.702, p < 0.001) with a within-subjects effect size of η_p_^2^ = 0.300. A contrast analysis revealed greater spectral magnitude in the numerosity condition than in all other conditions (size: F(1, 39) = 32.611, p < 0.001; spacing: F(1, 39) = 23.615, p < 0.001; random: F(1, 39) = 27.381, p < 0.001). The spectral magnitude for size and spacing conditions did not differ from the spectral magnitude for the random condition (p > 0.459).

### 3.2 Children’s SSVEP sensitivity to numerical, but not non-numerical, magnitude increases over development

#### 3.2.1 Behavioral results

The mean (±std) accuracy of the color judgment task was 0.777 (±0.160) and the mean (±std) response time was 971.4 (±259.6) ms.

#### 3.2.2 SSVEP response to the dot arrays in children

I first examined the 8-Hz spectral magnitude in children to identify channels that are maximally tagged by the flickering of the dot arrays. As shown in Fig. 3A, 8-Hz spectral magnitude peaked at channels Oz and O2′ (about 0.08 rad lateral to O2 in the standard 10–20 system). A two-way repeated-measures ANOVA with the four conditions (numerosity, size, spacing, and random) and the two channels (Oz and O2′) revealed non-significant interaction (F(3, 138) = 2.396, p = 0.077) and main effects (condition: F(3, 138) = 1.773, p = 0.163; channel: F(1, 46) = 0.130, p = 0.721), indicating that the amplitude spectra at the frequency (8 Hz) at which the dot arrays flickered was not affected by the different trial types. As in the case of adults, the two channels that peaked in 8-Hz spectral magnitude, Oz and O2′, were chosen for further investigations in the systematic variations in numerosity, size, and spacing, as reported in the following Section.

#### 3.2.3 SSVEP index of numerical sensitivity in children

SSVEP sensitivity to numerosity, size, and spacing across all children was quantified by the spectral magnitude at 1 Hz, and a repeated-measures ANOVA was used to test the effects of the four experimental conditions separately at Oz and O2′. At Oz, there were no significant differences in the 1-Hz spectral magnitude across the four conditions (F(3, 138) = 1.257, p = 0.292), as can be visually examined in Fig. 3B. In contrast, at O2′, there was a significant effect of condition (F(3, 138) = 11.703, p < 0.001) with a within-subjects effect size of η_p_^2^ = 0.203. A contrast analysis revealed greater spectral magnitude in the numerosity condition than in all other conditions (size: F(1, 46) = 16.065, p < 0.001; spacing: F(1, 46) = 26.473, p < 0.001; random: F(1, 46) = 15.470, p < 0.001). At the same time, the spectral magnitude for the size and spacing conditions was not significantly greater than the spectral magnitude for the random condition (p > 0.737). These results collectively indicate robust, selective sensitivity to numerical magnitude in children at least in the right occipital region.

#### 3.2.4 Age-related changes in the SSVEP index of numerical and non-numerical magnitudes

A multivariate linear regression analysis was used to quantify the association between the 1-Hz spectral magnitudes in the four conditions (as dependent variables) and children’s age (as an independent variable). This analysis revealed a statistically significant association between 1-Hz spectral magnitudes and age (F(4, 42) = 6.128, p = 0.001, Wilk’s Λ = 0.631, η_p_^2^ = 0.369). Note that a similar multivariate regression analysis with independent variables of age, sex, and age × sex was initially performed, but the two latter terms were not significant (p > 0.546) and were therefore dropped from the analysis.

**Fig. 4:**
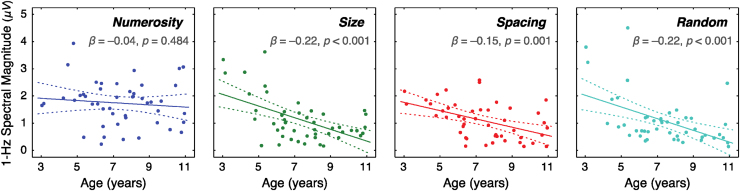
SSVEP power to systematic variations (1 Hz) in numerical (numerosity) and non-numerical (size and spacing) magnitudes as well as to random variations (baseline) as a function of age. 1-Hz spectral magnitude in the size and spacing conditions declined as a function of age similar to the decline of the baseline 1-Hz spectral magnitude, while 1-Hz spectral magnitude in the numerosity condition showed no such a pattern.

In order to better understand the results of this multivariate test, parameter estimates (slope) for the individual dependent variables were examined (Fig. 4). There was a non-significant effect of age on the SSVEP sensitivity to numerosity (β = −0.040, 95% CI = [−0.155 0.075], p = 0.484); however, there was a significant effect of age in the three other experimental conditions (size: β = −0.215, 95% CI = [−0.314 −0.115], p < 0.001; spacing: β = −0.152, 95% CI = [−0.238 −0.067], p = 0.001; random: β = −0.215, 95% CI = [-0.329 −0.102], p < 0.001). Note that this general pattern could not be explained by a small number of outlying data points. When the same regression analysis was conducted after removing relatively high influential observations (Cook’s d > 0.20), the same pattern was observed in which the effect of age was not present in the numerosity condition (p = 0.289) but was present in the three other conditions (p < 0.001). In order to examine the extent to which the youngest children influenced the overall pattern, two three-year-olds were further removed from the analysis. Yet, the same pattern was observed in which SSVEP sensitivity to numerosity was stable across age (p = 0.313) whereas SSVEP sensitivity to other conditions decreased as a function of age (p < 0.010).

Given that the slope was uniformly negative in the size, spacing, and random conditions but not in the numerosity condition, a posthoc contrast analysis was performed to test whether the age-related slope for the sensitivity to numerosity was different from the three other slopes using a bootstrapping method. Specifically, the observed linear contrast of the four slopes (weights of [+1 −1/3 −1/3 −1/3] for numerosity, size, spacing, and random, respectively) was tested against a simulated null distribution of this linear contrast by random relabeling of the experimental conditions 10,000 times. This analysis revealed that the age-related slope for the sensitivity to numerosity was significantly different from the three other slopes combined (p = 0.007). In sum, these results indicate that the trajectory of 1-Hz spectral magnitude for numerosity is substantially different from the trajectory of 1-Hz spectral magnitude for other magnitude dimensions as well as the baseline neural activity.

## 4 Discussion

In this study, a novel steady-state visual evoked potential (SSVEP) application was developed in order to quantify neural sensitivity to numerical and non-numerical dimensions of a dot array (Fig. 1). First, in adult participants, SSVEPs in the medial and right occipital (Oz & PO8′) sites were specifically sensitive to variations in numerosity (Fig. 2), consistent with previous results in ERPs (Park et al., 2016). Although the SSVEP measure does not carry temporal information, considering the ERP sensitivity to numerosity at Oz around 75 ms and near PO8 around 180 ms (Park et al., 2016), it is highly likely that the 1-Hz modulation at Oz is driven primarily by the oscillation of the C1 ERP component (Clark et al., 1994, Di Russo et al., 2002) and that the 1-Hz modulation at PO8′ is driven primarily by the oscillation of the N1 and P2 ERP components. Thus, the current SSVEPs in adults likely reflect selective modulation of early visual cortical activity by numerosity. It should be noted that the presentation of the dot arrays was very fast and there was no explicit task associated with the magnitude of the dot arrays. In fact, post-experiment debriefing in adults revealed that participants were completely ignorant about the experimental manipulation. Thus, it is unlikely that the current SSVEP patterns (i.e., selective sensitivity to numerosity) reflect any attentional or familiarity biases towards the numerosity dimension compared to other dimensions.

As in Park et al. (2016), SSVEP patterns in the current study are best and most parsimoniously explained by numerosity and not by other non-numerical magnitudes (individual area, total area, field area, sparsity, individual perimeter, total perimeter, coverage, and closeness). These results again suggest that there exists a neural mechanism for direct perception of numerosity. Such an interpretation may be incomprehensible to those who believe that non-numerical perceptual processes must take place prior to extracting numerosity information (Gebuis et al., 2016, Leibovich et al., 2016b). In fact, a few recent studies argue that neural signatures of numerosity processing are entirely driven by non-numerical cues (Gebuis and Reynvoet, 2012, Gebuis and Reynvoet, 2013, Soltesz and Szucs, 2014). I speculate that seemingly contradictory patterns between the current results and those previous studies arise from stimulus design. In an attempt to de-confound numerical and non-numerical cues of a dot array, those previous studies imposed a very strong nonlinear relationship between numerical and non-numerical cues, with a much larger variance in non-numerical cues and a comparatively smaller variance in numerosity across the stimuli used in their experiments (e.g., see Gebuis and Reynvoet, 2011). Such an approach is problematic at least for three reasons (also argued in Park et al., in press). First, a non-linear relationship between numerical and non-numerical cues and between various non-numerical cues makes it difficult, if not impossible, to assess the contribution of each of the dimensions in explaining neural data. Second, using dot arrays with a much larger variance in one dimension compare to another can mask the effect of the dimension with a smaller variance. In those previous studies, the variance in numerosity was almost always much smaller than the variance of other non-numerical dimensions. Third, such a previous approach is incapable of expressing which of the non-numerical dimensions maximally explain neural data. Because many different non-numerical dimensions exist and because their underlying neural computational mechanisms are presumably different from each other, it is insufficient as a hypothesis to claim that the processing of non-numerical cues as a whole is the basis of numerosity perception.

In contrast, the stimulus design used in this study along with the passive viewing paradigm exploiting rapid stimulus presentation makes it possible to evaluate the contribution of each of the numerical and non-numerical magnitude dimensions on the neural activity. Moreover, dot arrays spanned in equal ranges across various magnitude dimensions in this study, which enabled a fair comparison between the contributions of those magnitude dimensions. Using this innovative stimulus design, it is again found here that the visual cortex is selectively sensitive to numerosity. Sensitivity to numerosity, however, does not necessarily mean that the neural system, particularly in the early visual areas, is capable of the “summation” computation (see Dehaene and Changeux, Dehaene and Changeux, 1993; Verguts and Fias, 2004). Nonetheless, it is possible that early visual areas extract information of a visual scene while normalizing size and precise location (akin to the normalization layer in the computational model proposed by Dehaene and Changeux, 1993). In fact, it has recently been demonstrated that a deep network model with two hidden layers only to achieve efficient coding of sensory data (i.e., without any information about numerosity) successfully retrieves numerosity information of an image (Stoianov and Zorzi, 2012). That said, it is certainly plausible for early visual cortex to be encoding numerosity information as a statistical property of the visual scene.

More central to the objective of the study, I tested whether this specific neural sensitivity to numerosity exists in young children (3–10 years) and if so how it develops. The results first revealed that children’s SSVEPs do exhibit specific sensitivity to numerosity (Fig. 3). However, there were two notable differences between the adults and children’s SSVEP patterns. First, right occipital SSVEPs evoked by the dot array presentation (8 Hz) were more medial in children than in adults (compare Figs. 3A and 2A). Such a difference suggests that overall brain morphology and physiology (such as cortical folding patterns and myelination) continues to develop in late childhood and adolescence (between 10 and 18 years of age). Second, perhaps more interestingly, unlike adults, children did not show selective SSVEP modulation by numerosity in the medial occipital site (Oz) (see Fig. 3B). Given the results in adults (Fig. 2) in combination with previous ERP findings (Park et al., 2016), it is likely that children’s 8-Hz SSVEPs from Oz are also arising from the C1 ERP component. If so, the current pattern suggests that a neural mechanism for extracting numerical information very early in the visual cortical stream found in adults (Park et al., 2016) is devoid in children, or at least emerges much later in childhood. Consistent with this explanation, 1-Hz spectral magnitude for numerosity at Oz trended to be greater than those in other conditions when an older subset of children (≥9 years) were analyzed (vs. size: F(1, 18) = 5.248, p = 0.034; vs. spacing: F(1, 18) = 3.737, p = 0.069; vs. random: F(1, 18) = 4.574, p = 0.046). Because the functional role of this extremely early cortical visual processing stage is unknown, it is difficult to interpret the current group differences. Future studies should investigate the role of the early visual cortex in numerosity perception and the significance of its protracted development.

The innovative technique used in this study allowed an objective and independent assessment of children’s neural sensitivity to numerical and non-numerical magnitudes as a function of age. At the right occipital site (O2′), where selective sensitivity to numerosity was found in the group as a whole, sensitivity to non-numerical cues was negligible throughout development. To be specific, age-related changes in the SSVEPs in response to variations in size and spacing highly overlapped with age-related changes in the baseline SSVEPs (i.e., random variations). These results suggest an absence of neural mechanisms specifically responsive to non-numerical magnitudes across these ages tested in this study, at least in the early visual processing stage measured by the current SSVEP technique. Given that non-numerical cues significantly influence numerosity judgments in a numerosity comparison task both in adults and in children (Miller and Baker, 1968, Ginsburg and Nicholls, 1988, Allik and Tuulmets, 1993, Mix et al., 2002, Rousselle et al., 2004, Rousselle and Noël, 2008, Sophian and Chu, 2008, Soltesz et al., 2010, Nys and Content, 2012, Tibber et al., 2012, Defever et al., 2013, Fuhs and McNeil, 2013, Gilmore et al., 2013, Tokita and Ishiguchi, 2013, Leibovich et al., 2016a), the results suggest that non-numerical magnitudes are actively encoded much later in the visual stream or interferes with numerosity judgments at the stage of decision making. At the same time, these results reject the proposal that the representation of continuous, non-numerical magnitudes developmentally *precedes* the representation of discrete, numerical magnitudes (Mix et al., 2002, Leibovich and Henik, 2013).

In contrast to the SSVEP sensitivity to non-numerical magnitudes, neural sensitivity to numerosity increased gradually from 3 to 10 years of age. Note that the 1-Hz amplitude spectrum for numerosity actually maintained a flat slope across age. However, the baseline amplitude spectrum decreased continuously in childhood, which is a widely acknowledged phenomenon presumed to be influenced by changes in synaptic density, myelination, and skull thickness (Feinberg et al., 1990, DeBoer et al., 2007). From that perspective, the resulting pattern of non-decreasing neural sensitivity to numerosity is conceptually identical to increased sensitivity to numerosity as a function of age. This pattern of results demonstrating the existence of an early visual cortical mechanism for number perception was as hypothesized, based on the rationale that our sense of number is developmentally primitive. Furthermore, increasing neural sensitivity to numerosity between 3 and 10 years of age highly resembles the increase in children’s behavioral performance in discriminating numerosities in that same period (Halberda and Feigenson, 2008, Mussolin et al., 2012). It is worthy to note that non-symbolic numerical ability correlates with education level in people with very restricted numerical lexicon, thereby highlighting the role of education in the development of the approximate number system (Piazza et al., 2013). However, most of the effects were driven by adult participants in that previous work, and no study to my knowledge has directly tested that hypothesis in very young children. That said, it is certainly an open possibility that some intrinsic sensory and cognitive maturation may be driving the development of children’s non-symbolic numerical abilities. Therefore, together with putative later cognitive and decision-making stages for numerosity estimation, an early perceptual mechanism for numerosity found in the present study may serve as a basis for the approximate number system.

Conversely, the developmental trajectory points out that the effect of selective sensitivity for numerosity is very weak, if not absent, in the youngest children. Quantitatively speaking, a 95% prediction interval for a hypothetical 3-year-old child’s SSVEP sensitivity to numerosity was [1.36 2.47], overlapping substantially with the prediction intervals for size [1.58 2.54], for spacing [1.35 2.18], and random [1.47 2.58] conditions (see Fig. 4). This pattern of results was surprising given that even infants are known to attend to numerical features over non-numerical cues (Xu and Spelke, 2000, Lipton and Spelke, 2003, Libertus and Brannon, 2010 but see counterarguments in Mix et al., 2002). This pattern may indicate that younger children indeed do not have a robust early perceptual mechanism for processing discrete magnitude as argued in Mix et al. (2002), although current data do not show evidence for the existence of a visual mechanism for non-numerical magnitude either. According to this interpretation, very young children and infants may rely on other neural substrates besides the early visual cortical mechanism to discriminate numerosity (e.g., Collins et al., 2017 demonstrate the role of the subcortex in numerosity perception). Alternatively, subtle effects in selective neural sensitivity to numerosity may have been masked by relatively high statistical noise due to fewer sampling in the youngest children in this study. Future studies should test the mechanisms of direct perception of numerosity in very young children and infants for a clearer explanation.

The present study provides a methodological innovation by introducing SSVEPs in assessing numerical and non-numerical quantity processing. SSVEPs have high signal-to-noise ratio and are robust to artifacts (Vialatte et al., 2010), making them reliable measures. One previous study used SSVEPs to investigate the neural oscillatory responses to numerosity in infants (Libertus et al., 2011). Their use of the SSVEP approach, however, was different from the present approach. In that study, the same dot array image flickered at a fixed rate, and the spectral magnitude for that frequency was compared to the power of the same frequency induced by the flickering of another image with either more or less dots. Thus, unlike the current approach, multiple trial types were necessary to quantify the effect of a single dimension in that study. Furthermore, although Libertus et al. (2011) attempted to control for non-numerical effects (e.g., by equating non-numerical cues between sets of dot array images), without a systematic manipulation of these non-numerical cues it remains questionable whether the estimated SSVEPs solely represented the effect of numerical cues and to what extent the spectral magnitude was contaminated by non-numerical effects. Here, in the present study, non-numerical effects were not controlled for; rather they were actively estimated, which were later parsed out when estimating the effects of numerosity.

Whether the present SSVEP approach is more or less reliable than the previous ERP approach is an empirical question. In the previous work (Park et al., 2016), a regression model was used to estimate the effects of the three orthogonal dimensions (numerosity, size, and spacing) in explaining the ERPs. I performed a reliability analysis of that previous dataset by estimating beta coefficients (of numerosity, size, and spacing) in each experimental block of Exp. 1 in that study and then by computing Cronbach’s alpha on the averaged beta (i.e., the mean of *β_numerosity_*, *β_size_*, and *β_spacing_*). Across the three channels of interest in previous work (Park et al., 2016), Cronbach’s alpha ranged from 0.32 to 0.47. I then computed the Cronbach’s alpha from the current SSVEP dataset in adults by computing the averaged 1-Hz spectral magnitude (across the numerosity, size, and spacing conditions) in each experimental block. It was around 0.92. These results indicate that the present SSVEP approach does yield a more reliable measure of neural sensitivity to numerical and non-numerical magnitudes of a dot array, hence making the SSVEP measure a more appropriate index for studying individual differences and changes.

In sum, the newly developed SSVEP technique allowed an estimation of neural sensitivity to numerical and non-numerical dimensions of a dot array in adults and children. In adults, a robust SSVEP effect of numerosity but negligible effects of non-numerical magnitudes were observed in the medial and right occipitoparietal channels, largely replicating previous ERP findings (Park et al., 2016). More importantly, this selective neural sensitivity to numerosity was found in young children (although exclusively in the right occipitoparietal site), demonstrating the existence of a neural mechanism for direct perception of number at young ages. Furthermore, this selective neural sensitivity to numerosity emerged gradually from near non-existence in three-year olds to strong sensitivity in ten-year olds, potentially providing a neural mechanistic explanation for the development of humans’ primitive, non-verbal ability to comprehend number.

## Conflict of interest

None.
